# A Massive Overdose of Dalfampridine

**DOI:** 10.5811/westjem.2015.8.26127

**Published:** 2015-12-03

**Authors:** Laura J. Fil, Payal Sud, Steven Sattler

**Affiliations:** * North Shore University Hospital, Department of Emergency Medicine, Manhasset, New York; † Long Island Jewish Medical Center, Department of Emergency Medicine, New Hyde Park, New York; ‡ Good Samaritan Hospital, Department of Emergency Medicine, West Islip, New York

## Abstract

Multiple sclerosis (MS) is an immune mediated inflammatory disease that attacks myelinated axons in the central nervous system. Dalfampridine (4-aminopyridine) was approved by the Food and Drug Administration in January 2010 for treatment of MS. Our patient was a 34-year-old male with a history of MS, who was brought to the emergency department after being found unresponsive. His current medications were valacyclovir, temazepam, dalfampridine (4-AP) and a tysabri intravenous (IV) infusion. Fifteen minutes after arrival the patient seized. The seizures were refractory to benzodiazepines, barbiturates and phenytoin. The 4-AP level was 530ng/mL (25ng/mL and 49ng/mL). The patient stopped seizing on hospital day 3 and was discharged 14 days later with normal mental status and neurologic exam. 4-AP is a potassium channel blocker that blocks the potassium ion current of repolarization following an action potential. The blockade of the potassium channel at the level of the membrane widens the action potential and enhances the release of acetylcholine, thus increasing post-synaptic action potentials. The treatment of patients with 4-AP overdose is supportive. Animal data suggest that patients with toxic levels of 4-AP may respond to phenytoin. Our case illustrates the highest recorded level of 4-AP in an overdose. Our patient appeared to be refractory to a combination of high doses of anticonvulsants and only improved with time.

## INTRODUCTION

Multiple sclerosis (MS) is an immune-mediated inflammatory disease that attacks myelinated axons in the central nervous system. It is characterized by short-term episodes of neurologic deficits that usually resolve completely or almost completely. Patients with MS may suffer from pain due to spasticity, fatigue and progressive disability.^1^ There is currently no cure for the disease, but a plethora of treatments are available and are continuously being developed.

One of the more novel medications on the market is dalfampridine, or 4-aminopyridine (4-AP). 4-AP was originally developed to be used as an avicide. The chemical was studied in mammals and was shown to cause hyperexcitability, hypersalivation, tremors, muscle incoordination, convulsions, cardiac or respiratory distress.^2^

4-AP acts as a voltage-gated potassium channel blocker that blocks the potassium ion current of repolarization in an axon following an action potential. Potassium efflux out of the axon is the ionic mechanism that results in repolarization of the axon. Repolarization must occur before the axon can generate another action potential. In MS, the myelin sheath is thinned and this results in either a weakened signal when the action potential reaches its destination or a complete block. This leads to weakness and fatigability of strength.^3^ The blockade of the potassium channel widens the action potential, and thus 4-AP increases the conduction of action potentials and this leads to increased strength.^2^ Due to the ability of 4-AP to reverse synaptic blockade it has been used as a treatment in many different neuromuscular disorders such as Alzheimer’s disease, botulism, Eaton Lambert syndrome and multiple sclerosis.^4^ In January 2010, 4-AP was approved by the FDA in the United States under the name dalfampridine to help patients with MS improve their gait and strength when walking.^5^

There are always new medications being developed for the treatment of MS, a disease with many potential comorbidities; among them, depression is the most notable. This occurs in part because MS is a chronic disease and also there are theories that it occurs because of frontal or subcortical white matter disease.^6^ Here we present a case of a 4-AP overdose with the highest reported level in the literature.

## CASE REPORT

A 34-year-old male with a past medical history of MS was brought into the emergency department by Emergency Medical Services after being found unresponsive by his mother with three pill bottles at his side. The bottles were an empty bottle of valacyclovir, an empty bottle of temazepam and a bottle of dalfampridine still containing some pills. His mother gave the history that the patient was under multiple social stressors of late. Other than MS, the patient was diagnosed with insomnia and had no other pertinent past medical history. His current medications were valacyclovir, temazepam, dalfampridine (4-AP) and a tysabri IV infusion (all part of his MS treatment regimen).

The initial vital signs were as follows: Blood pressure: 155/82mmHg; Heart rate: 106/min; Respiratory rate: 24/min; Temperature: 97.4ºF; O_2_ sat: 97% 2L NC. The bedside glucose was 144mg/dl. The patient appeared extremely tremulous, was awake, but not responding to questioning nor following simple commands. He responded to tactile stimulation by localizing to the pain. He did not appear to have any focal deficits. His pupils were 4 mm, equal and reactive and his mucous membranes were moist. His heart sounds were tachycardic, with no murmurs. The patient had clear breath sounds bilaterally and no retractions. The abdomen had normoactive bowel sounds. The skin was diaphoretic with piloerection.

Intravenous access was established, labs were drawn and the patient was given two liters of normal saline. Soon after arrival, the patient lost consciousness, his oxygen saturation decreased to below 90% and he began to have a tonic-clonic seizure. The patient was administered boluses of lorazepam, to a total of 8 mg, without effect in seizure resolution. He was intubated and subsequently placed on a lorazepam and propofol infusion for sedation. He was loaded with one gram of phenytoin and 300mg phenobarbital and then placed on a phenobarbital infusion. Clinically the patient continued to have frequent, recurrent seizures.

The patients lab values on arrival were significant for a white blood cell count of 31.1×10^3^/mcL, a sodium of 143mEq/L, potassium 3.1mEq/L, chloride 107mEq/L, bicarbonate of 24mEq/L, a blood urea nitrogen of 15mg/dL and a creatinine of 1.2mg/dL. The anion gap was 12. The lactic acid was 3.4mg/dl. Total creatinine kinase was 99 units/L. Liver function tests and coagulation studies were all within normal limits. Ethanol, acetaminophen and salicylate levels were all negative. A urine toxicology screen was positive for benzodiazepines. An arterial blood gas showed pH 7.22, pCO_2_ 65, pO_2_ 127 and HCO_3_ 26.6 (performed immediately after intubation). Computerized tomography of the brain and chest x-ray were both normal. The electrocardiogram showed a sinus rhythm at 88 beats per minute with normal QRS and QT intervals. Drug levels were sent for 4-AP and valacyclovir. The 4-AP level resulted at 530ng/mL (therapeutic: 25 to 49ng/mL). The valacyclovir level was 7.5mcg/mL (therapeutic 2.0–4.0mcg/mL).

The patient was admitted to the intensive care unit (ICU) and while he was there he continued to have seizures. While in the ICU the patient had two electroencephalographs (EEGs) performed. The EEG on hospital day 2 showed epileptiform activity and the other EEG was performed on hospital day 7 and was indicative of epileptiform encephalopathy.

The patient stopped seizing on hospital day 3. The patient was extubated on hospital day 12. He was discharged, with normal mental status and neurologic exam to an inpatient psychiatric facility.

## DISCUSSION

Due to the fact that 4-AP is not a commonly used medication, not much is known about how it acts at toxic levels in humans. Van Diemen et al. looked at the dosage and serum level related to efficacy and safety. During this study a maximum daily dose of 0.5mg/kg body weight was not surpassed in any patient. Patients in this study experienced dizziness, paresthesias and restlessness at levels approaching 0.5mg/kg. No patient in the study had a seizure, as the level at which seizures usually occur is 0.8mg/kg body weight.^7^

4-AP acts as a potassium channel blocker that prolongs the action potential, thus increasing neuromuscular activity. It is theorized that the increase in neuromuscular activity is therapeutic at appropriate levels, but that the same mechanism causes adverse reactions at elevated levels.^8^ The adverse reactions are not only of the central nervous system, but also include injury to the cardiac and skeletal muscle. One study showed that 4-AP toxicity could result in permanent damage to the hippocampus and Papez circuit affecting memory and learning.^9^

The 4-AP level of our patient resulted at 530ng/mL. Until now, the highest documented 4-AP level published was 233.6ng/mL.^10^ In that case, the patient was treated with lorazepam boluses and the seizures subsided, as opposed to our patient who was refractory to treatment with multiple anti-convulsant medications. The valacyclovir level was also elevated and valacyclovir itself can be associated with neuropsychiatric symptoms. Seizures associated with valacyclovir are not well reported in the literature, and the few case reports on valacyclovir associated seizures are in patients with renal failure, which our patient did not have.^11^,^12^

The current treatment of 4-AP overdose is supportive. Animal data suggests that patients with 4-AP toxicity will respond to phenytoin. It is hypothesized that because phenytoin acts as a sodium channel blocker it will inhibit action potentials from continuing.^9^ A study by Yamaguchi et al.^13^ showed that treatment of mice with elevated levels of 4-AP with gamma butyric acid (GABA) was comparable to other phenytoin-like therapies. Our patient did not respond to treatment with phenytoin. This raises the question of whether or not a second phenytoin load should be used, or additional anti-convulsants that have a similar mechanism of action to phenytoin should be added.

Using phenytoin to treat seizures secondary to 4-AP is an important concept because phenytoin is not usually part of the algorithm for the treatment of toxin-induced seizures. The recommendation for treating an undifferentiated toxin-induced seizure is to use a benzodiazepine or barbiturate, then pyridoxine and finally propofol.^14^ Toxin-induced seizures differ from seizures from other causes because they usually occur due to a global lowering of the seizure threshold in previously normal neurons, whereas seizures due to epilepsy occur in a focal lesion of abnormal neurons. Phenytoin is effective for patients with idiopathic seizure disorders or with defined structural or electrical foci of seizure activity, as phenytoin prevents the propagation of seizures (include reference). There are many reports of phenytoin used in toxin-induced seizures causing clinical detriment to the patient.^14^

This case illustrates the highest recorded level of 4-AP in an overdose. Optimal therapy for this overdose is unknown. Our patient appeared to be refractory to a combination of high dose intravenous benzodiazepines, phenytoin, propofol and phenobarbital and only improved with time.
